# Differential Programming of B Cells in AID Deficient Mice

**DOI:** 10.1371/journal.pone.0069815

**Published:** 2013-07-29

**Authors:** Marc A. Hogenbirk, Marinus R. Heideman, Arno Velds, Paul CM. van den Berk, Ron M. Kerkhoven, Bas van Steensel, Heinz Jacobs

**Affiliations:** 1 Division of Biological Stress Response, The Netherlands Cancer Institute, Amsterdam, The Netherlands; 2 Genomic Core Facility, The Netherlands Cancer Institute, Amsterdam, The Netherlands; 3 Division of Gene Regulation, The Netherlands Cancer Institute, Amsterdam, The Netherlands; 4 Friedrich Miescher Institute, Basel, Switzerland; Chang Gung University, Taiwan

## Abstract

The *Aicda* locus encodes the activation induced cytidine deaminase (AID) and is highly expressed in germinal center (GC) B cells to initiate somatic hypermutation (SHM) and class switch recombination (CSR) of immunoglobulin (Ig) genes. Besides these Ig specific activities in B cells, AID has been implicated in active DNA demethylation in non-B cell systems. We here determined a potential role of AID as an epigenetic eraser and transcriptional regulator in B cells. RNA-Seq on different B cell subsets revealed that *Aicda^−/−^* B cells are developmentally affected. However as shown by RNA-Seq, MethylCap-Seq, and SNP analysis these transcriptome alterations may not relate to AID, but alternatively to a CBA mouse strain derived region around the targeted *Aicda* locus. These unexpected confounding parameters provide alternative, AID-independent interpretations on genotype-phenotype correlations previously reported in numerous studies on AID using the *Aicda^−/−^* mouse strain.

## Introduction

During B cell development the GC cycle is initiated when mature B cells contact with cognate follicular helper T cells within secondary lymphoid organs. Extensive proliferation of antigen-primed B cells causes secondary follicles to polarize and form two micro-anatomically distinct regions within the GC, the T cell zone-proximal dark zone (DZ) and the distal light zone (LZ). In the DZ, rapidly proliferating B cells (centroblasts (CB)) can change the genetic code of antibodies due to the initiation of two independent processes: somatic hypermutation (SHM) and class switch recombination (CSR). Exit of the centroblast from the cell cycle coincides with the relocation of non-cycling GC B cells (centrocytes (CC)) to the LZ. The LZ contains antigen specific follicular helper T cells and networks of follicular dendritic cells. The latter are coated with immune-complexes. CCs continuously scan these coated follicular dendritic cells to test their variant B cell receptors for antigen binding ability. Eventually, these cells differentiate into memory B cells to establish immunological memory or plasma B cells to ascertain effective immunity. Survivors of the GC reaction express the appropriate antibody class and bind antigen with higher affinity [1]. The observation that the vast majority of mature B cell lymphomas arise from GC implies that B cells undergoing the GC reaction are at high risk for oncogenic transformation [2].

The crucial finding, that both SHM and CSR require the activity of AID led to the first profound insights into the molecular mechanism of these processes [3], [4]. AID is a member of the *Apobec* gene family of cytosine deaminases [5], [6], [7]. AID binds ssDNA and preferentially deaminates cytosine residues that reside within the WRC motif [8], [9], [10]. During SHM, AID deaminates cytosines within rearranged V(D)J segments that encode the variable domain of Ig heavy and light chains. Subsequent processing of the uracil involves error prone DNA repair enabling the introduction of somatic mutations at a rate approximating one point mutation per generation. This process eventually leads to formation of high affinity antibody variants. To initiate CSR, AID deaminates C in the top and bottom strands of two transcriptionally active S regions. To generate DNA double strand breaks (DSBs) in switch regions, the uracil has to be processed by components of the base excision repair or mismatch repair system. Once the DSBs are generated, the intervening DNA fragment is deleted, and the downstream constant region is juxtaposed to the upstream variable region. This process enables B cells to change their antibody isotype and adapt the effector function of the antibody [11]. The majority of the AID pool resides cytosolic and only a small fraction is actively shuttled between cytosol and nucleus, which is one of several strategies to control its mutagenic potential [4].

Studies in non-B cell systems implicate a role for AID in active CpG demethylation [12], [13], [14], [15], [16], [17]. DNA demethylation controls biological functions like changes in gene expression and chromatin organization to orchestrate cellular differentiation. In addition, DNA methylation contributes to genome stability and is a hallmark off X chromosome inactivation in females. Reprogramming of hetereokaryons was proposed to require AID-dependent DNA demethylation of the *Oct4* and *Nanog* promoters [14]. In primordial germ cells genome-wide AID-dependent DNA demethylation was proposed to occur in exons, introns and intergenic regions but not in promoters. This study further favored the view that AID targets genome-wide and functions as an epigenetic regulator [15]. The possibility that AID exerts an additional function as an epigenetic eraser in GC B cells, in which AID expression is highest, has not been tested to date.

In some B lymphoid cancers translocation breakpoints found in or near switch regions implicated AID in stimulating ectopic chromosomal translocations. Besides the scheduled AID-dependent DSBs in switch regions, AID is implicated in generating DSBs also in non-Ig genes [18], [19], [20]. High-Throughput, Genome-wide Translocation Sequencing (HTGTS) and Translocation Capture sequencing (TC-seq) studies suggest that AID may be required to induce DSBs in ∼120 genes leading to chromosomal translocations [21], [22]. Further indirect evidence for AID ‘off-targeting’ was provided by somatic mutation analysis in TSS-proximal regions. The authors estimate that 25% of expressed genes in germinal center B cells are deaminated by AID [23]. Of note, conclusions on potential genome-wide impact of AID are heavily based on a single *Aicda^−/−^* mouse model [3], which was generated in the embryonic stem cell line TT2 derived from F1 C57BL/6 x CBA blastocysts [24]. So far, in depth validations of this mouse model by next-generation technologies are lacking.

To study the potential role of AID in B cell programming and tumor development, we here applied MethylCap [25], [26] and RNA-Seq [27] on different B cell subsets. Our analyses provide alternative AID-independent interpretations on genotype-phenotype correlations previously observed in numerous studies on AID using the *Aicda^−/−^* mice.

## Results

### *Aicda^−/−^* GC B Cells have a CC Signature

**Figure 1:**
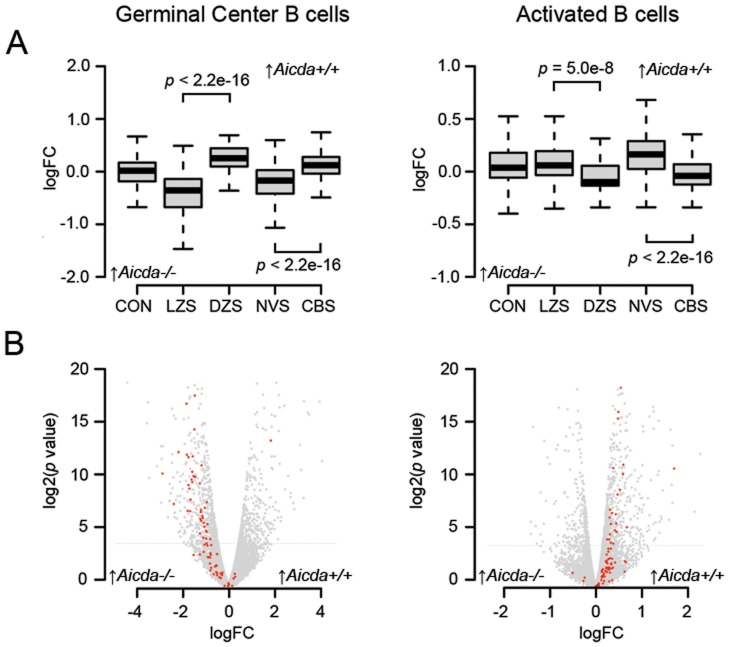
Transcriptome comparisons of GC B cells and *in vitro* activated B cells from *Aicda^+/+^* and *Aicda^−/−^* mice. A) Box-plot of previously defined gene groups which are differentially expressed between *Aicda^+/+^* and *Aicda^−/−^* GC (left panel) and activated (right panel) B cells: CON, control group; LZS light zone signature genes [29], DZS, dark zone signature genes [29]; NVS, naïve B cell signature genes [28], CBS, centroblast signature genes [28]. For statistical analysis the sign test was applied. B) Volcano-plot of genes differentially expressed between *Aicda^+/+^* and *Aicda^−/−^* GC (left panel) and activated (right panel) B cells. The *Ighv* genes are shown in red. For statistical analysis the sign test was applied.

Active DNA demethylation by AID has previously been implicated in gene regulation of non-B cell systems [13], [14], [15]. To explore the possibility that AID alters transcription in B cells to control B cell differentiation we applied RNA-Seq on CD19^+^/CD95^+^/PNA^hi^ GC B cells, isolated from spleens of immunized *Aicda^−/−^* and *Aicda^+/+^* mice. The expression of 155 genes (FDR <0.01) was found to differ between *Aicda^−/−^* and *Aicda^+/+^* GC B cells (Table S1 & Figure S1 and S2). GO analysis indicated six major categories: cellular response, signaling, regulation, homeostasis, differentiation/development, and cellular activation (Figure S3A and S3B). To further pinpoint the phenotype of *Aicda^−/−^* GC B cells, we took advantage of two previously defined gene groups that distinguish naïve B cells from CBs [28] and DZ B cells (CB) from LZ (CC) B cells [29]. When plotting the differential gene expression distribution of each gene group individually, we revealed that GC B cells of *Aicda^−/−^* mice have a more pronounced CC signature (Figure 1A; left panel). *Igh* transcription increases at the transition from CB to CC. Consistent with this notion, *Ighv* transcripts are more abundant in GC B cells of *Aicda^−/−^* mice (Figure 1B; left panel).

### *In vitro* Activated *Aicda^−/−^* B Cells have a CB Signature

The *in vivo* results not necessarily relate to AID since failure to undergo SHM and CSR may affect the composition of CD19^+^/CD95^+^/PNA^hi^ GC B cells such that CC are overrepresented within enlarged GC of *Aicda^−/−^* mice [3]. To identify B cell intrinsic transcriptional differences, we applied RNA-Seq on *in vitro* activated B cells isolated from *Aicda^−/−^* and *Aicda^+/+^* mice. This approach revealed that 145 genes differed in their expression (FDR cut-off at 0.01) (Table S1 and Figure S2B). GO analyses indicated that these genes fall into the same six GO categories (Figure S3C). The assessment of the CB and CC gene signatures revealed that *in vitro* activated B cells of *Aicda^−/−^* mice acquire a CB like signature (Figure 1A, right panel). As *Igh* transcription decreases at the transition from naïve to CB stage, one expects that *Ighv* transcripts are less abundant in activated *Aicda^−/−^* B cells, which indeed was the case (Figure 1B, right panel).

### Methyl-Cap does not Reveal AID-dependent CpG Demethylation

**Figure 2:**
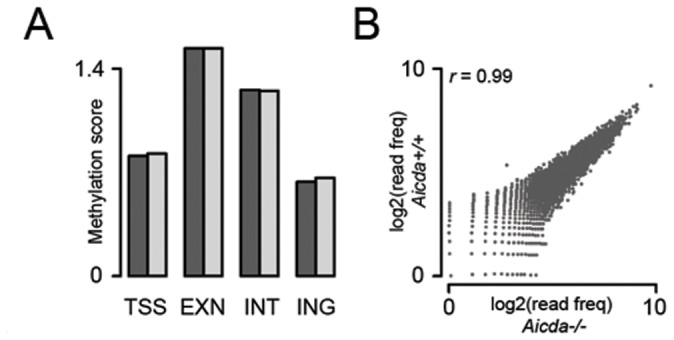
Assessment of AID-dependent DNA demethylation. A) Comparisons of CpG methylation load between *Aicda^+/+^* and *Aicda^−/−^* GC B cells in defined genomic elements. TSS, transcription start sides (TSS); EXN, exons; INT, introns; ING, intergenic regions are shown. Various statistical tests revealed no differences. B) Correlation plot of MethylCap data obtained from *Aicda^+/+^* and *Aicda^−/−^* GC B cells. The correlation coefficient, *r* was determined applying the Persons’ test.

As AID has been proposed to affect the transcriptional program via deamination of methylated CpGs in specific promoters of hetereokaryons [14] and the methylome in exons, introns, and intergenic regions, but not in promoters of primordial germ cells [15], we argued that AID may affect the GC reaction by active CpG demethylation. To test this possibility we applied MethylCap-Seq on GC (CD19^+^/CD95^+^/PNA^hi^) B cells from immunized *Aicda^−/−^* and *Aicda^+/+^* C57BL/6 mice. MethylCap takes advantage of a high affinity methylbinding domain [25], [26] which when coupled to paramagnetic beads enables an efficient enrichment of methylated DNA fragments. Although MethylCap did enrich effectively for methylated fragments (Figure S4), the methylation load in promoters, exons, introns and intergenic regions remained indistinguishable between *Aicda^−/−^* and *Aicda^+/+^* GC B cells (Figure 2A). The methylation load of individual TSSs did not differ as well (Figure 2b). Apparently, MethylCap-Seq failed to reveal AID-dependent CpG demethylation.

### Naïve *Aicda^−/−^* B Cells are Pre-activated

**Figure 3:**
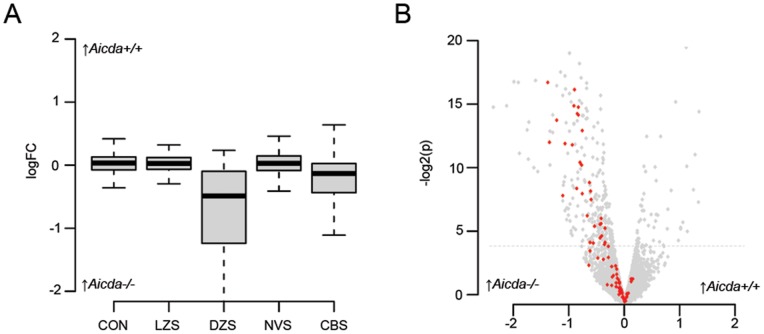
Naïve B cells are pre-activated. A) Box-plot of previously defined gene groups differentially expressed between *Aicda^+/+^* and *Aicda^−/−^* naive B cells: CON, control group; LZS light zone signature genes [29], DZS, dark zone signature genes [29]; NVS, naïve B cell signature genes [28], CBS, centroblast signature genes [28]. B) Volcano-plot of genes differentially expressed between *Aicda^+/+^* and *Aicda^−/−^* naïve B cells. The DZS genes are shown in red.

To further assess transcriptional differences between *Aicda^−/−^* and *Aicda^+/+^* mice, we isolated naïve CD19^+^/CD43^−^ B cells. As revealed by RNA-Seq, naïve B cells from *Aicda^−/−^* and *Aicda^+/+^* mice, in which AID is not expressed, differ substantially (Figure 3). DZ signature genes are expressed at higher levels in *Aicda^−/−^* naïve B cells (Figure 3A and 3B). Furthermore, GO-analysis revealed GO-terms linked to cell cycling (Figure S3D). These analyses revealed that naïve *Aicda^−/−^* B cells are pre-activated when compared to naïve *Aicda^+/+^* B cells and extend on previous observations made [3].

### *Aicda^−/−^* Mice are not Congenic

**Figure 4:**
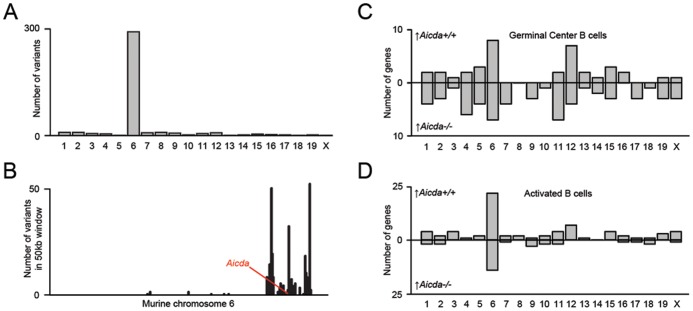
*Aicda^−/−^* mice are not congenic and *Aicda^−/−^* B cells are transcriptionally deregulated. A) CBA derived SNPs reside in chromosome 6. B) Number of variants per 50kb window is plotted against the position on chromosome 6, where *Aicda* (indicated in red) is located. CBA derived SNPs accumulate in a specific region. C) Contribution of each chromosome to the top 100 of differentially expressed genes between *Aicda^+/+^* and *Aicda^−/−^* GC B cells. The *chi* square test revealed that differential expressed genes located on chromosome 6 are significantly (p<2.2e-16) enriched in the region were CBA SNPs were found (see figure 4B, Table S1). D) As ‘C’, but now on activated *Aicda^+/+^* and *Aicda^−/−^* B cells. The *chi* square test revealed that differential expressed genes located on chromosome 6 are significantly (p<2.2e−16) enriched in the region were CBA SNPs were found (see figure 4B, Table S1).

As an alternative to AID in causing the transcriptional differences observed between *Aicda^−/−^* and *Aicda^+/+^* B cells, we considered artifacts related to gene targeting in ES cells as potential confounders for genotype-phenotype alterations previously observed when using these mice. *Aicda^−/−^* mice originate from the embryonic stem cell line TT2, which were derived from F1 C57BL/6 x CBA blastocysts [24]. In these TT2 cells the *Aicda* locus was targeted conventionally by replacing exon 3 and partially exon 4 encoding the cytidine-deaminase domain of AID with a NeoR selection cassette. This likely explains the observation of non-functional *Aicda* transcripts in naïve *Aicda^−/−^* B cells (Table S1). Chimeras were backcrossed to C57BL/6 mice. To determine whether the C57BL/6- or CBA-allele was targeted we performed SNP analysis. SNP analysis revealed that the CBA allele was targeted (Figure 4A and 4B). Moreover, the CBA derived region around the targeted *Aicda* locus persisted even after extensive backcrossing for 15 generations. To assess whether this region is also transcriptionally affected we analyzed the transcriptome of the various B cell subsets. In all subsets, this region (Chr6:E2-G1) always contributed most to the top hundred differentially expressed genes (Figure 4C and 4D; Table S1). The fact that genes within this region are up- as well as downregulated implies a deregulated region, where potentially due to strain differences, genetic or epigenetic alterations during ES cell culturing, and conventional gene targeting, wide ranging transcriptional alterations arise in *cis* and in *trans* (Table S1). Being aware of these major confounding variables, direct attributions to AID-specific functions cannot be based solely on genotype-phenotype correlations when using these mice.

## Discussion

This study was initiated to address the potential impact of AID on gene regulation of B cells. To address the potential role of AID in B cell programming, we applied RNA-Seq on diverse B cell subsets. RNA-Seq analysis revealed major differences between the transcriptomes of GC B cells from *Aicda^−/−^* and *Aicda^+/+^* mice. CC-specific transcripts were clearly overrepresented in GC B cells of *Aicda^−/−^* mice. The observation that GC B cells of *Aicda^−/−^* mice are enriched for CC-specific transcripts suggests that CCs accumulate in GC of *Aicda^−/−^* mice. In addition, major alterations in the transcriptome were also observed when comparing *in vitro* activated B cells from *Aicda^−/−^* and *Aicda^+/+^* mice, indicating major intrinsic differences between these activated B cells. Interestingly, *in vitro* activated *Aicda^−/−^* B cells acquire already a CB like gene signature. This signature likely relates to the fact that even naïve B cells from *Aicda^−/−^* are pre-activated. These findings are consistent with previous notions that *Aicda^−/−^* mice and patients suffering from the autosomal recessive form of Hyper-IGM syndrome (HIGM2) have enlarged GCs and hyper-activated B cells [3], [30]. Lack of AID activity suppresses DNA damage responses and maintains antigen-reactivity of GC B cells, which is likely to provide a selective advantage to AID deficient B cells. A defective GC reaction will delay antigen clearance and prolong immune activation, which in turn may break tolerance and stimulate the generation of autoreactive antibodies in mice and patients lacking AID [31], [32], with wide ranging consequences on adaptive immunity, including B cell development and the establishment of peripheral and central B cell tolerance. Therefore, in contrast to previous notions, an indirect AID-dependent effect on central and peripheral B cell tolerance cannot be excluded. The pre-activated status of ‘naïve’ B cells in *Aicda^−/−^* mice may also provide a cell-autonomous, AID-independent, preferential outgrowth of *Aicda^−/−^* B cells in bone marrow chimeric mice [31], [32].

The dramatic transcriptional alterations observed in different *Aicda^−/−^* B cell subsets let us to explore whether AID exerts a function in gene regulation by CpG demethylation as previously proposed for non-B cells [13], [14], [15]. Methyl-Cap did not reveal AID-dependent demethylation, neither region-specifically nor generically in GC B cells where AID is highly abundant. However, given the sensitivity of MethylCap, we cannot exclude the possibility that subtle differences in CpG methylation may have escaped detection. Notably, these results are consistent with biochemical insights demonstrating that *in vitro* AID has a strong preference in deaminating cytosine rather than methyl-cytosine [33], [34].

Most unexpectedly, *Aicda^−/−^* mice are not congenic. Based on SNP analysis, the CBA-derived *Aicda* allele was inactivated in TT2 ES cells. Surprisingly, the remaining CBA fragment around the targeted *Aicda* locus was differentially regulated throughout B cell development. Genes within this region significantly contributed to the top hundred of most differentially expressed genes found between naïve, activated and GC B cell subsets derived from *Aicda^−/−^* and *Aicda^+/+^* mice. Besides strain-specific differences, other genetic as well as epigenetic changes may have been acquired during gene targeting or maintaining T2 ES cells. This observation is not a specific feature of our mouse cohort, as these mice were backcrossed for 15 generations, which extensively exceeds the conventional number of backcrosses. As many studies heavily (or solely) rely on this unique *Aicda^−/−^* strain, these confounding variables add an unexpected level of complexity, which has not been considered before.

While it would be of interest how exactly the targeting affected gene regulation around the AID locus, such experiments require a heroic effort that unlikely contributes to our understanding about AID biology. Investing selective candidate genes, which could be responsible for the phenotypes, comes down to a ‘fishing expedition in deep water’ with partial or no phenocopy. The massive transcriptional alterations seen may relate to the CBA derived fragment (in *cis*), which may have systemic impact *in trans*. Furthermore, we cannot exclude genetic and epigenetic alterations that arose during ES cell culturing in this region. Given the long distance changes, the mere presence of a NeoR selection cassette is unlikely the driving force behind these drastic transcriptional alterations on chromosome 6. The finding that *Aicda^−/−^* mice carry a CBA-derived fragment in their genome argues that direct phenotype-genotype correlations in *Aicda^−/−^* mice cannot be made and consequently, additional validations are required to distinguish between AID-independent and dependent effects when comparing *Aicda^−/−^* and *Aicda^+/+^* mice.

However, one confounding issue may relate to *Mbd4*, a DNA glycosylase encoding gene implicated in active DNA demethylation [13]. *Mbd4* is encoded within the CBA region of chromosome 6 and is differentially expressed in *Aicda^−/−^* B cell subsets. Provided that *Mbd4* has a function in active DNA demethylation, this might have influenced previous methylation differences measured in gene bodies between primordial germ cells *of Aicda^−/−^* and *Aicda^+/+^* mice [13], [15]. Clearly, the scope of this study is not to validate retrospectively the contribution of these complex confounding variables to previous conclusions made in numerous AID-studies. To distinguish easily between AID-dependent and confounding, i.e. AID-independent issues in the future, the generation of a novel, conditional, congenic *Aicda−/−* strain using the Cre/loxP recombination system in C57BL/6 derived ES cells is likely the most straight forward solutions in solving these problems.

In conclusion, while transcriptional differences were observed between *Aicda^−/−^* and *Aicda^+/+^* B cells, MethylCap and SNP analyses question whether these differences merely relate to the presence or absence of AID. Confounding parameters related to conventional gene-targeting in mice may have major implications on previous genotype/phenotype comparisons, where conclusions were based on the assumption that differences between *Aicda^−/−^* and *Aicda^+/+^* B cells only relate to the mere absence of AID.

## Materials and Methods

### Mice

*Aicda^−/−^* mice were kindly provided by T. Honjo and backcrossed for 15 generations to C57BL/6. All experiments were approved by an independent animal ethic committee of the Netherlands Cancer Institute and executed according to national guidelines.

### Isolation of B Cell Subsets

CD43 expressing cells were depleted from splenic single-cell suspensions of 2-mo-old *Aicda^−/−^* and *Aicda^+/+^* mice using paramagnetic streptavidin-beads and biotinylated anti-CD43. From the remaining cells a fraction was used to sort naïve B cells (CD19^+^, IgM^+^, CD43^−^, DAPI^−^). From the other fraction *in vitro* activated B cells were derived by stimulation with LPS [20 µg/ml] and IL-4 (10% of supernatant of IL-4 producing cells). *In vitro* activated B cells (CD19^+^, DAPI^−^) were sorted four days after culturing. To isolate GC B cells, 2-mo-old *Aicda^−/−^* and *Aicda^+/+^* mice were immunized by a single intraperitoneal injection of 10^8^ sheep red blood cells in 200 µl HBSS. Ten days later, GC B cells (CD19^+^, PNA^high^, CD95^+^, DAPI^−^) were sorted from splenic single-cell suspensions.

### Cells

Human embryonic kidney cells (HEK 293T) and Ramos cells (Burkitt’s lymphoma) were maintained in IMDM (Gibco) supplemented with 8% FCS and antibiotics. 3T3-NTZ indicator cells [35] were cultured in DMEM supplemented with 8% FCS and antibiotics.

### Methyl-Cap

The MethylCap protocol (Diagenode) and sequence library preperation (Illumina) were performed according to manufacturers protocol.

### RNA-Seq

TruSeq RNA sample preparation and subsequent library preparation was according to manufacturers protocol (Illumina).

### Experimental Design, Analysis and Statistical Testing

Each *Aicda^−/−^* (2x) and *Aicda^+/+^* (2x) GC B cell library (MethylCap and RNA-Seq), originates from pooled lymphocytes of at least 3 mice. For RNA-Seq on *Aicda^−/−^* (4x) and *Aicda^+/+^* (4x) naïve B cells, and *Aicda^−/−^* (4x) and *Aicda^+/+^* (4x) activated B cells, a total of sixteen libraries were individually prepared and indexed for each individual mouse. For all experiments it holds that the complete procedures were repeated on different days to generate true biological replicates. For further quality checks, and statistical testing, we used the R packages *limma*, *edgeR* and *GoSeq* [36], [37]. For GO visualization we used with the enrichment map plugin of *cytoscape2*. For SNP analyses variants were called between *Aicda^−/−^* and *Aicda^+/+^* with *somatic sniper*, intersected with cds.gtf (ucsc), and coding variants are linked to Sanger SNP file. For further analysis we used the R language (http://www.r-project.org/).

### Data Accession

RNA-Seq and MethylCap data deposited under accession number GSE47705.

## Supporting Information

Figure S1 **Experimental design.** Each *Aicda^−/−^* (2x) and *Aicda^+/+^* (2x) GC B cell library (MethylCap and RNA-Seq), originates from pooled lymphocytes of at least 3 mice. For RNA-Seq on *Aicda^−/−^* (4x) and *Aicda^+/+^* (4x) naïve B cells, and *Aicda^−/−^* (4x) and *Aicda^+/+^* (4x) activated B cells, a total of sixteen libraries were individually prepared and indexed for each individual mouse. For all experiments it holds that the complete procedures were repeated on different days to generate true biological replicates.
(TIF)

Figure S2 **Assessment of the reproducibility of RNAseq data.** A) MA-plot of *Aicda^−/−^* and *Aicda^+/+^* GC B cells (left panel). The top 100 differentially expressed genes, higher in the *Aicda^+/+^* (red) of replicate 1, are indicated in replicate 2 (red). Differentially expressed genes with a FDR <0.1 after combining datasets (*edgeR* [*36*]) are indicated in blue. B) Same as in ‘A’, but activated B cells. C) MA-plot of *Aicda^−/−^* and *Aicda^+/+^* Naive B cells. The top 100 differentially expressed genes, higher in the *Aicda^−/−^* of replicate 1, are indicated in replicate 2.
(TIF)

Figure S3 **GO-analysis.** A) GO-terms differential between *Aicda^−/−^* and *Aicda^+/+^* GC B cells as unbiased identified by *GOseq* and clustered by *cytoscape2*. B) As, in ‘A’. GO-groups were as follows: cellular response (I), signalling (II), regulation (III), homeostasis (IV), differentiation/development (V), and cellular activation (VI). C) GO-groups differential between *Aicda^−/−^* and *Aicda^+/+^* activated B cells. Same group definition as in ‘b’. D) GO-groups differential between *Aicda^−/−^* and *Aicda^+/+^* naïve B cells. GO-groups were as follows: deamination (VII) and cell cycle(VIII).
(TIF)

Figure S4 **Enrichment of methylated DNA fragments from B cells.** Pie charts reveal specific enrichment of hypermethylated *Tsh2* and hypomethylated *Gapdh* genes in the bound fractions and flow through fraction, respectively. The quantitative PCR was performed according to manufacturers protocol.
(TIF)

Table S1 (XLS)
