# Supplementary figures and images for: Differential Programming of B Cells in AID Deficient Mice
Source: PLoS One. 2013 Jul 29;8(7):e69815. doi: 10.1371/journal.pone.0069815 (PMC3726761; doi:10.1371/journal.pone.0069815)

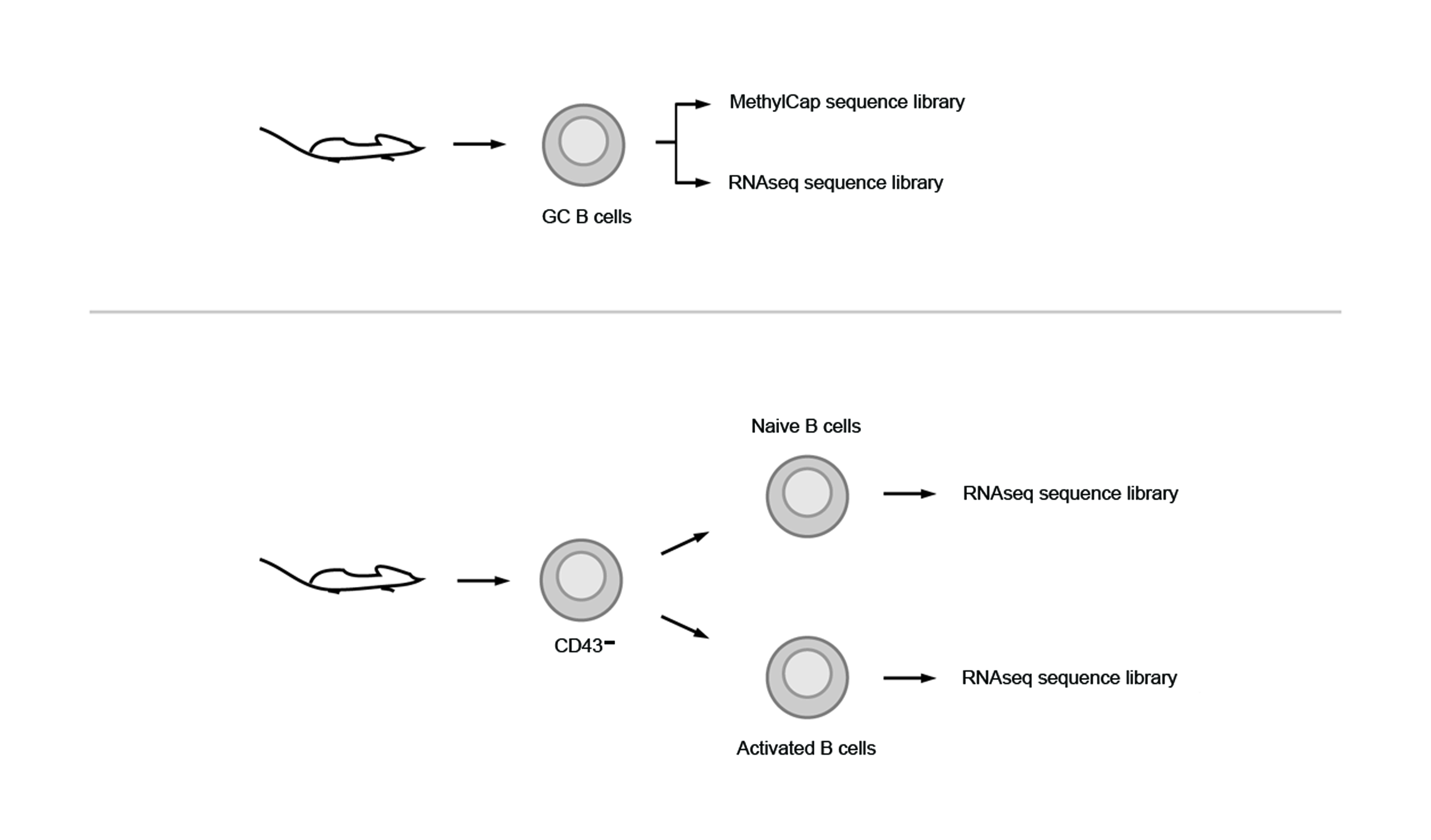

Supplement: Figure S1 — Experimental design. Each Aicda−/− (2x) and Aicda+/+ (2x) GC B cell library (MethylCap and RNA-Seq), originates from pooled lymphocytes of at least 3 mice. For RNA-Seq on Aicda−/− (4x) and Aicda+/+ (4x) naïve B cells, and Aicda−/− (4x) and Aicda+/+ (4x) activated B cells, a total of sixteen libraries were individually prepared and indexed for each individual mouse. For all experiments it holds that the complete procedures were repeated on different days to generate true biological replicates. (TIF) [file pone.0069815.s001.tif]

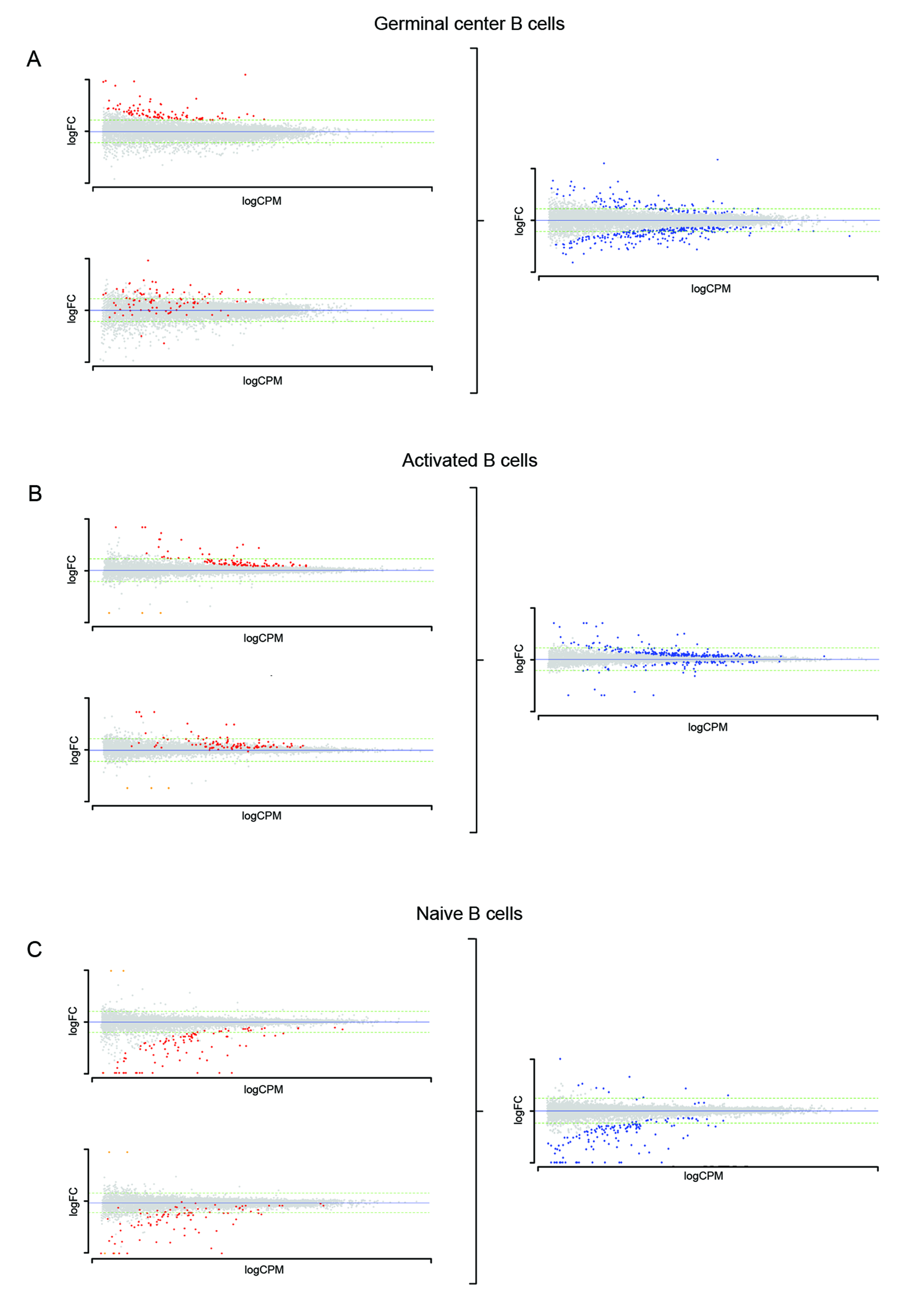

Supplement: Figure S2 — Assessment of the reproducibility of RNAseq data. A) MA-plot of Aicda−/− and Aicda+/+ GC B cells (left panel). The top 100 differentially expressed genes, higher in the Aicda+/+ (red) of replicate 1, are indicated in replicate 2 (red). Differentially expressed genes with a FDR <0.1 after combining datasets (edgeR [36]) are indicated in blue. B) Same as in ‘A’, but activated B cells. C) MA-plot of Aicda−/− and Aicda+/+ Naive B cells. The top 100 differentially expressed genes, higher in the Aicda−/− of replicate 1, are indicated in replicate 2. (TIF) [file pone.0069815.s002.tif]

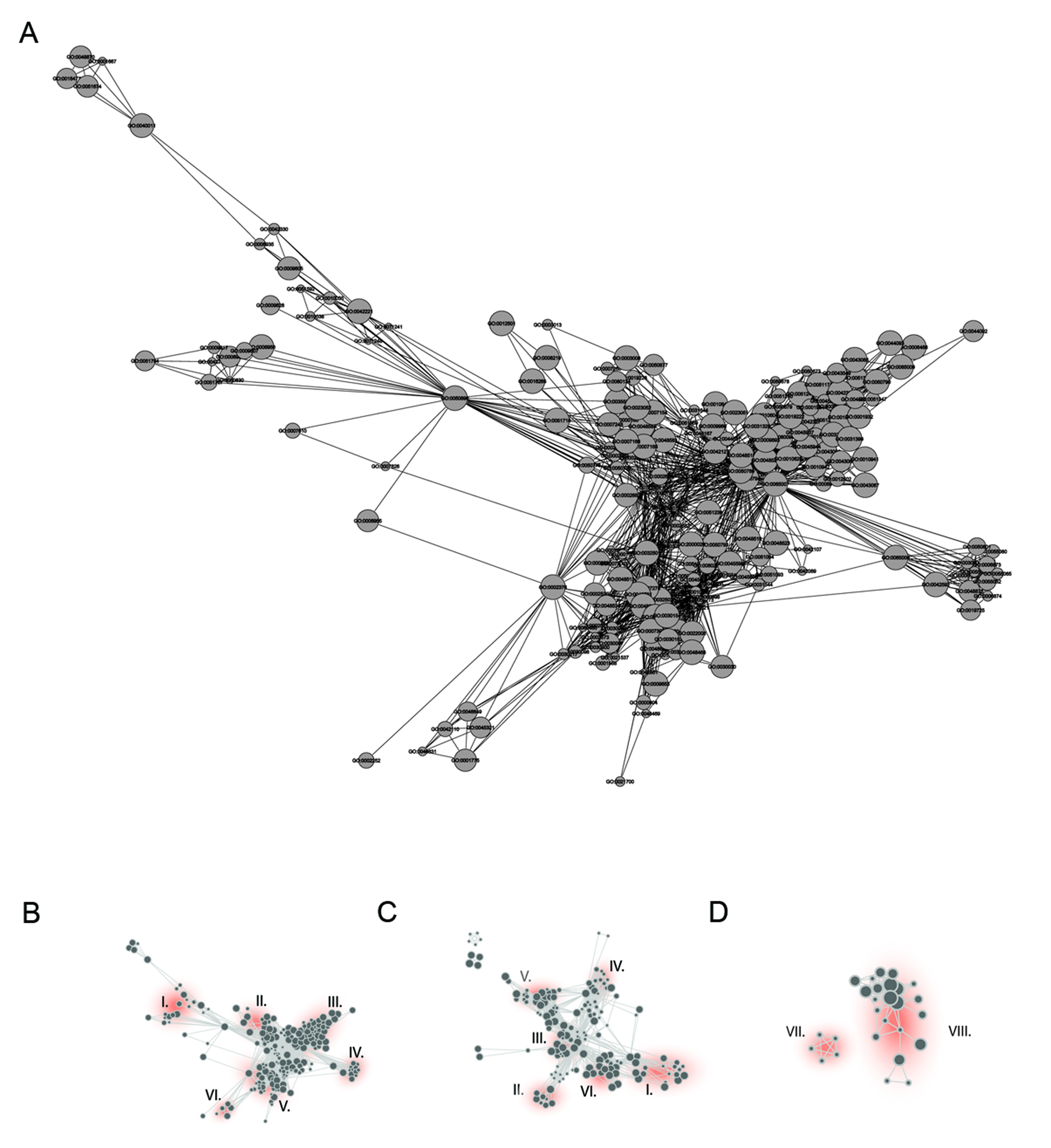

Supplement: Figure S3 — GO-analysis. A) GO-terms differential between Aicda−/− and Aicda+/+ GC B cells as unbiased identified by GOseq and clustered by cytoscape2. B) As, in ‘A’. GO-groups were as follows: cellular response (I), signalling (II), regulation (III), homeostasis (IV), differentiation/development (V), and cellular activation (VI). C) GO-groups differential between Aicda−/− and Aicda+/+ activated B cells. Same group definition as in ‘b’. D) GO-groups differential between Aicda−/− and Aicda+/+ naïve B cells. GO-groups were as follows: deamination (VII) and cell cycle(VIII). (TIF) [file pone.0069815.s003.tif]

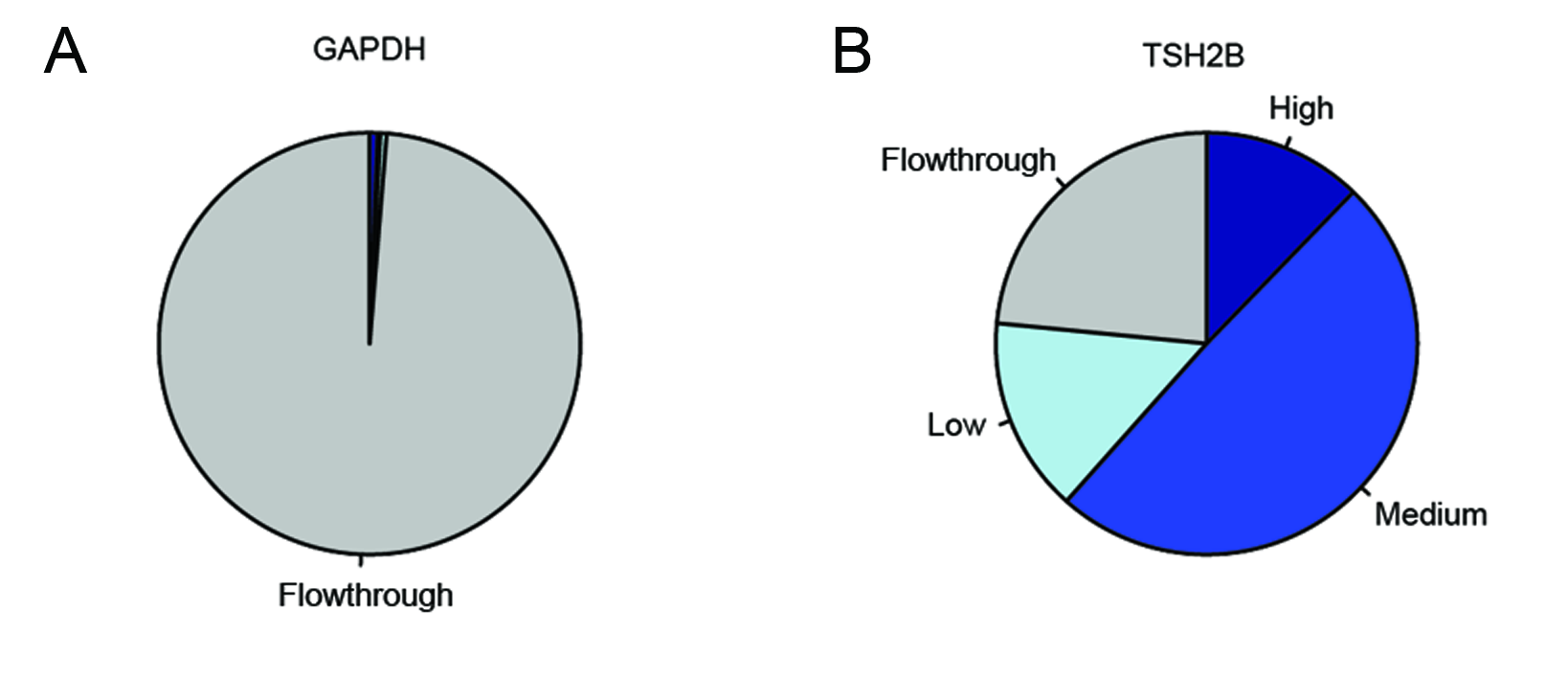

Supplement: Figure S4 — Enrichment of methylated DNA fragments from B cells. Pie charts reveal specific enrichment of hypermethylated Tsh2 and hypomethylated Gapdh genes in the bound fractions and flow through fraction, respectively. The quantitative PCR was performed according to manufacturers protocol. (TIF) [file pone.0069815.s004.tif]
